# Paramedian transparietal approach to the lateral ventricle in a dominant hemisphere: how I do it

**DOI:** 10.1007/s00701-024-06382-7

**Published:** 2024-12-03

**Authors:** Simone Grannò, Abdullah Al Awadhi, Adrien May, Philippe Bijlenga

**Affiliations:** Division of Neurosurgery, Department of Clinical Neurosciences, Geneva University Hospitals, Geneva, Switzerland

## Abstract

**Background:**

Intraventricular tumors present significant surgical challenges due to their deep location and proximity to critical neuroanatomical structures. Surgical strategies include the transtemporal, interhemispheric and transparietal approaches, each carrying specific risks. Recently, a paramedian transparietal approach to a lateral ventricle meningioma in the dominant hemisphere was described.

**Methods:**

We propose a mini-craniotomy, mixed reality-assisted paramedian transparietal approach via a trans-sulcal route for safe tumor resection while preserving critical white matter tracts.

**Conclusions:**

This technique enhances the safety and efficacy of intraventricular tumor resection, particularly in young patients with dominant hemisphere lesions.

**Supplementary Information:**

The online version contains supplementary material available at 10.1007/s00701-024-06382-7.

## Introduction

The successful resection of atrial intraventricular tumors poses a unique operative challenge in neurosurgery [10]. Their deep positioning within the eloquent brain, close proximity to crucial perforating arteries and envelopment by multidimensional white matter tracts can be technically daunting. When localised to the dominant hemisphere, these lesions carry an even greater risk of post-operative neurological sequelae [8].

Intraventricular tumors are rare and large, typically found in the atria of the lateral ventricles [3]. They usually include meningiomas, ependymomas, or gliomas [10]. These tumors grow slowly and are initially asymptomatic but can rapidly lead to life-threatening hydrocephalus and mass effect.

Historically, surgical resection was associated with high morbidity and mortality [8, 10]. However, advancements in neurosurgical techniques, planning and neuronavigation have significantly improved outcomes [3, 5, 10].

### Relevant surgical anatomy

Three primary operative techniques are known: the transtemporal, interhemispheric transcallosal, and oblique transparietal approaches [4, 6, 7, 9].

The transtemporal approach is the most direct, allowing early access to the tumor’s choroidal vessels without excessive lesion mobilisation [1, 3]. However, tumor-specific displacement of vascular structures may limit this benefit. Additionally, this route is generally too risky for tumors in the dominant hemisphere due to involvement of the posterior temporal cortex and the optic radiation, superior longitudinal fasciculus (SLF), and arcuate fasciculus (AF).

The interhemispheric transcallosal approach, described by Kempe and Blaylock [7], minimises cortical disruption and spares the optic radiation, SLF, and AF. However, it is inherently trans-forniceal and requires sectioning the splenium of the corpus callosum, leading to potential memory impairment, disconnection syndromes, spatial orientation issues, and emotional processing effects [6].

The transparietal approach offers a compromise, avoiding the optic radiation, SLF, and AF, but with later vascular access. This approach can result in post-operative Gerstmann syndrome for dominant hemisphere lesions. A superolateral-oblique trajectory has been proposed to minimise the risk of injuring the superior sagittal sinus [4, 5].

A 2021 case report by Andrews et al. [1] proposed a *paramedian* transparietal approach with a nearly vertical, line-of-sight corridor. The authors resected an atrial intraventricular meningioma in the dominant hemisphere of a 55-year-old female, utilising fibre tracking and standard neuronavigation. The patient was positioned on a head frame in a neutral angle with a slight chin tuck. Their proposed approach required a large, inverted-C incision and an extended square craniotomy with four burr holes.

Here, we propose a *mini-craniotomy* variant of the paramedian transparietal approach, requiring a single midline incision. We combine preoperative DTI MRI planning with our knowledge of intraoperative navigation in mixed reality [2] to achieve a cortically conservative corridor with minimal dural opening.

### Clinical presentation

**Fig. 1:**
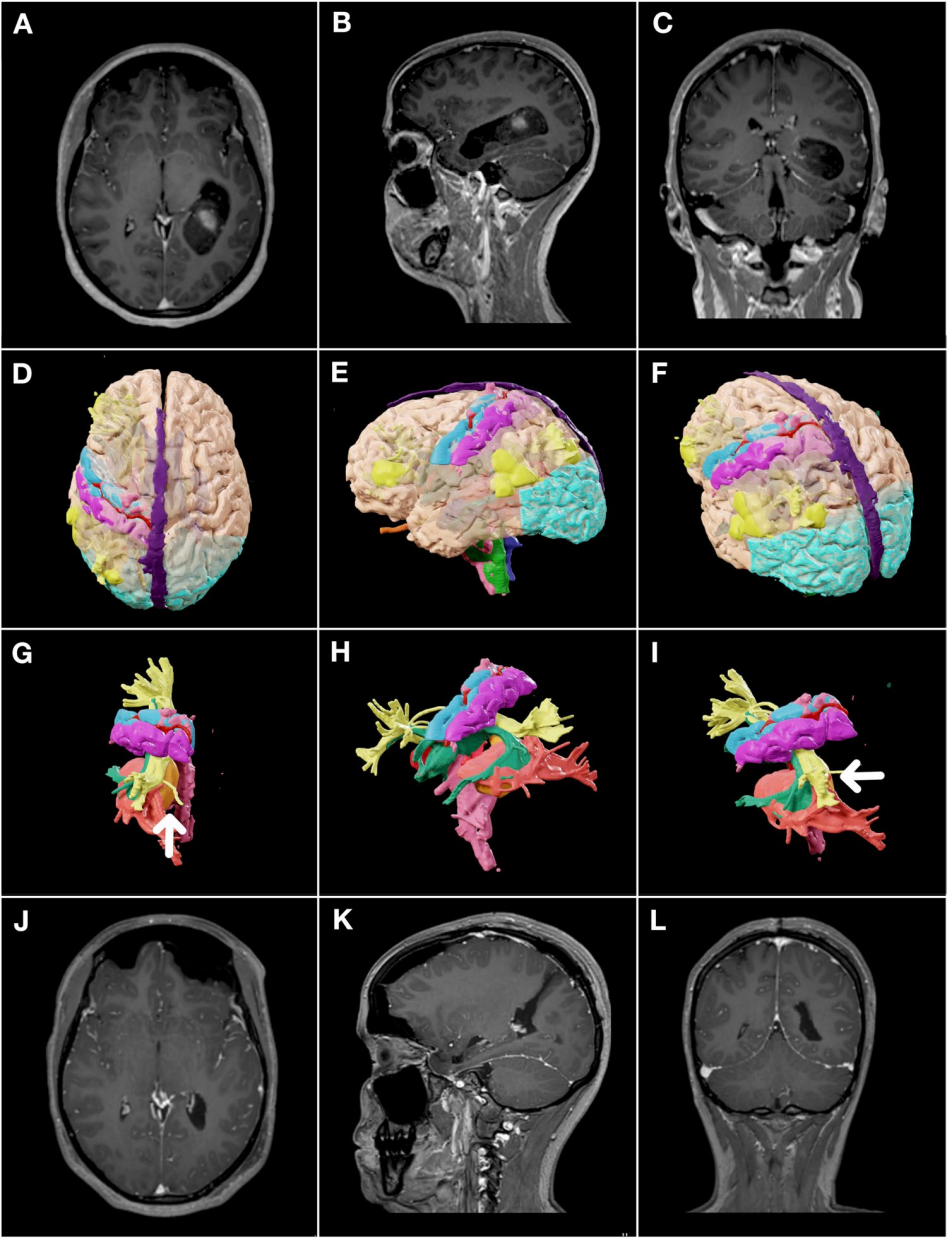
Peri-operative imaging and surgical planning. Contrast T1 MRI in the axial, sagittal and coronal planes pre (**A-C**) and post (**J-L**) operatively. **D-I** − 3D reconstruction of DTI fibre tracking in iPLanNet. Orange: lesion. Light red: optic radiation and lateral geniculate nucleus. Yellow: superior longitudinal fasciculus. Green: arcuate fasciculus. Light blue: precentral gyrus. Magenta: postcentral gyrus. Turquoise: occipital lobe. **G -** surgical line-of-sight view. **H** - sagittal view. **I -** Oblique view

A previously healthy, left-handed 22-year-old female presented with intermittent diurnal headaches and occasional blurred vision in the right eye. Contrast MRI revealed a 45 × 28 mm intraventricular lesion in the left temporal horn and atrium (Fig. 1A), causing ventricular dilation and moderate uncal herniation. Clinical examination was unremarkable at GCS 15/15, no cranial nerve or focal neurological deficits. Neuropsychological assessment identified anterograde semantic memory deficits and right-sided hemineglect. Functional MRI showed left-sided language activation, ipsilateral to the lesion. No intracranial hypertension was noted.

## Description of the technique

### Pre-operative planning

We employed DTI MRI with fibre tracking for 3D reconstruction in iPlanNet (BrainLAB AG, Germany) for intraoperative navigation (Fig. 1 D). The reconstruction identified the neuroanatomical boundaries of the tumor. The lesion (Fig. 1G, orange) is bordered by the optic radiation and lateral geniculate nucleus (light red) anterolaterally, and superolaterally by the SLF and AF (yellow and green). The precentral and postcentral gyri (light blue and magenta) with their descending fibers lie anterolaterally, and Wernicke’s area is inferolateral due to left-sided language activation.

Given these vital boundaries, a narrow transparietal corridor was thus triangulated (Fig. 1G, I, white arrow), granting direct line-of-sight access to the lesion (Fig. 1G orange, surgical view). This aligns with Andrews et al., where a transtemporal approach was obstructed by critical white matter tracts, and an oblique transparietal approach was limited due to the postcentral gyrus proximity. The reconstruction was converted to mixed-reality overlays [2] for microsurgery.

### Patient positioning and craniotomy

**Fig. 2:**
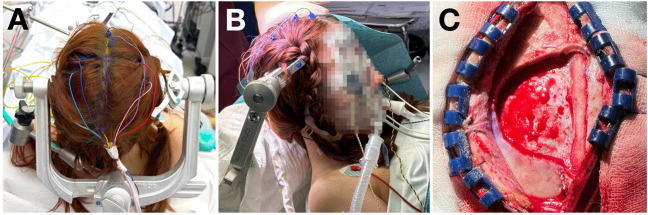
Patient positioning and craniotomy. **A-B** - Head in the neutral position with a chin tuck. The midline incision is marked in blue in panel A. **C** - Minicraniotomy extending 5 cm laterally from the midline to allow paramedian access to the transparietal corridor

The head was fixed neutrally, slightly elevated with a chin tuck (Fig. 2A). Neuromonitoring electrodes were placed for sensorimotor, language, and visual function (Fig. 2A). For a more conservative approach an 8-cm midline incision was performed (Fig. 2C). Two burr holes, 5 cm apart, were drilled over the superior sagittal sinus. A semicircular mini-craniotomy was carried out with a 5 cm radius.

### Microsurgical resection in mixed reality

**Fig. 3:**
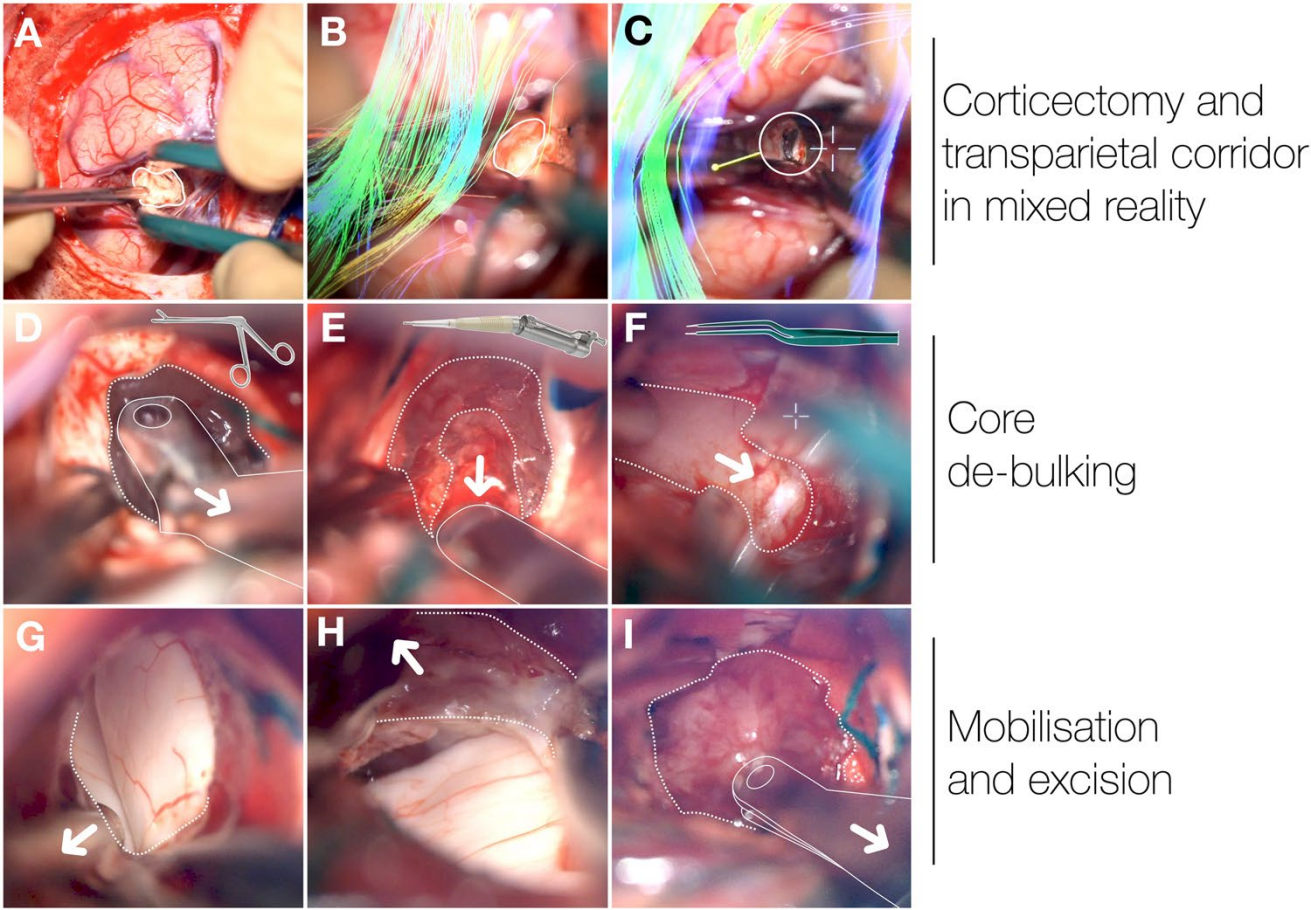
Overview of the surgical procedure. **A** - Paramedian corticectomy on the left superior parietal lobule (solid line). **B** - Extension of the 10 mm paramedian transparietal corridor under mixed reality control. **C** - Approach to the lateral ventricular wall and initial visualisation of the lesion (circle). **D** - Initial mobilisation and core de-bulking of the tumor (dotted line) by microsurgical rongeur (solid line). **E** - Main tumor de-bulking by ultrasonic aspirator (solid line). **F** - Infero-medial mobilisation, cauterisation and detachment of the tumor. **G** - Infero-lateral mobilisation and detachment of the tumor, visualisation of the parahippocampal gyrus. **H** - Supero-lateral mobilisation and detachment of the tumor, visualisation of the hippocampus. **I** - En bloc excision of the tumor by microsurgical rongeur (solid line)

A sharp trans-sulcal dissection was performed through the intraparietal sulcus (Fig. 3A) reaching its depth at 25 mm from the dura mater. Thereafter, a corticectomy was performed. Using a mixed reality overlay, we then established a paramedian transparietal corridor (Fig. 3B, white line) bordering the optic radiation, AF, and SLF (Fig. 3B, overlays). The corridor was extended approximately 10 mm through the cortex and white matter until reaching the lateral ventricle as the lesion came into view (Fig. 3C, circle).

The tumor (Fig. 3D, dotted line) was initially dissected with a microsurgical rongeur (straight line) and then de-bulked using an ultrasonic aspirator (Fig. 3E). De-vascularisation was achieved with a bipolar cautery. Mixed reality and axial MRI confirmed alignment with the lesion’s core.

The tumor was mobilised inferomedially, inferolaterally, and superolaterally (Fig. 3F, dotted lines and arrows), cauterising its attachments until the parahippocampal gyrus (Fig. 3G) and hippocampus (Fig. 3H) were revealed. The lesion was then removed en bloc by rongeur (Fig. 3I). A final mixed reality check confirmed sparing of critical fibers. There were no intraoperative complications. The total operating time was 4 h and 50 min, the microsurgical segment lasting 3 h and 30 min.

### Postoperative course

The patient exhibited no cranial nerve, visual, sensorimotor, or language deficits. Detailed examination revealed fluctuating finger agnosia and mixed agraphia, consistent with a mild, incomplete Gerstmann syndrome. Anterograde semantic memory loss and right-sided hemineglect, known pre-operatively, persisted. Neuropsychological assessment suggested a good prognosis for functional recovery, recommending a short outpatient rehabilitation cycle. Postoperative MRI confirmed complete lesion resection without complications (Fig. 1, J-L). Pathology identified a low-grade glioma, requiring no further treatment. The patient was reassessed at 3 and 6 month from surgery and made an excellent recovery with no remaining deficits other than mild attentional dysfunction.

### Limitations and how to avoid complications

All patients should receive DTI MRI with fibre tracking, allowing for the precise identification of critical neuroanatomical boundaries and the establishment of safe, patient-tailored surgical corridors in mixed reality [2]. Neuro-ophthalmological examination should be performed to account for any pre-operative visual deficits, as there is a risk of injuring important visual pathways. In left-handed patients, language fMRI is crucial to establish whether a lesion is in the dominant hemisphere.

Protecting the brain parenchyma intraoperatively is paramount. Employing a mini-craniotomy reduces exposure. Maintaining a line-of-sight view through optimal head positioning can prevent off-target lesions. We apply Merocel™ pads directly over brain tissue in order to keep it humid (removed in the video for demonstrative purposes). CSF can be drained early on by opening the arachnoid and keeping the partial pressure pCO_2_ at 4.5 kPa to reduce swelling. Traumatic manipulation of the parenchyma is minimised by avoiding the use of retractors as much as possible (used only to prevent collapsing of the opening from gravity), preferring the application of small folded humid cotton pads, that are kept later unfolded by aspirating the fluids, making them slightly more rigid.

Anticipating potential risks, such as Gerstmann syndrome, particularly in the dominant hemisphere, is crucial. Discussing these with the patient helps managing expectations and planning rehabilitation. Peri-operative neuropsychological assessment is highly recommended, and a low threshold for rehabilitation referral should be maintained, especially in young patients.

Limitations should be accounted for. The operative trajectories need to be planned in a 3D environment, requiring 20 min to an hour of work by a trained surgeon. Additionally, the surgeon needs to be proficient in the use of mixed reality navigation, which requires prior training. Challenges include accurately judging the depth when working in mixed reality with the microscope and correcting for any intraoperative shift of the mixed reality overlay. Both challenges can be securely overcome if standard operating procedures are trained and adopted [2]. Any missteps in this regard might lead to off-target resection and unintended brain matter lesions.

## Supplementary Information

Below is the link to the electronic supplementary material.

ESM 1 (MP4 229 MB)

## Data Availability

The clinical and imaging data discussed in this paper, including all pre-operative planning are protected by patient confidentiality and not openly available, but can be supplied in anonymised form upon reasonable request to the corresponding author. Data are located in controlled access data storage at Geneva university Hospitals.
